# Intraosseous Mucoepidermoid Carcinoma in the Mandible: A Report of Two Cases and Literature Review

**DOI:** 10.7759/cureus.73143

**Published:** 2024-11-06

**Authors:** Abid Majeed, Alok K Sethi, Damodar Bhat, Ashok k Jena, Pritinanda Mishra

**Affiliations:** 1 Department of Dentistry, All India Institute of Medical Sciences, Bhubaneswar, Bhubaneswar, IND; 2 Department of Pathology, All India Institute of Medical Sciences, Bhubaneswar, Bhubaneswar, IND

**Keywords:** intraosseous mucoepidermoid carcinoma mandible, intraosseous salivary gland tumor, malignant salivary gland tumor, mandible bone tumor, mucoepidermoid carcinoma (mec)

## Abstract

Mucoepidermoid carcinoma (MEC) is the most common malignant tumor of salivary glands.It usually occurs in major salivary glands. The occurrence of intraosseous MEC (IMEC) is rare and is usually seen in the mandibular premolar and molar regions.These tumors present mixed radiolucent and radiopaque images on radiographs, which makes the diagnosis difficult. Thus, histopathological evaluation is the definitive and accurate method for its diagnosis. The treatment depends on the histopathological grading of the tumors. Low-grade tumors are usually treated with radical resection surgeries, and high-grade ones need definitive radiotherapy in addition to radical resection surgeries and neck dissection. Intraosseous involvement of MEC in the mandible is rare. This manuscript highlights the clinical presentation and management of two cases of IMEC of the mandible.

## Introduction

Mucoepidermoid carcinoma (MEC) is the most common malignant tumor of the salivary gland. It comprises about 5-10% of all salivary gland malignancies [1,2]. It usually occurs in major salivary glands like parotid, submandibular, and sublingual glands, but it can also occur in minor glands present in the palate, retro-molar area, and jaw bones [2]. Apart from the head and neck region, mucoepidermoid carcinoma can also occur in breasts [1]. Intraosseous mucoepidermoid carcinoma (IMEC) comprises about 2-3% of all head and neck MEC and is usually seen in the mandibular premolar and molar region [1]. Stewart, in 1945, described it as a distant clinical entity consisting of mucous and epidermal cellular elements [3]. It is commonly found in the 4th-5th decade of life with more predilection in females than males [4]. The leading cause of occurrence is the presence of an ectopic salivary gland in the bone, the transformation of cystic linings, or the intraosseous extension of submucosal salivary glands [1,2,5,6]. Intraosseous MEC, being a mixed radiolucent and radiopaque lesion, is barely being diagnosed on radiographs. Thus, histopathological evaluation is the definitive and accurate method for its diagnosis [4]. On the basis of histological features, these tumors are classified as low, intermediate, and high grade. The treatment usually depends on the histopathological grading of the tumors. Low-grade tumors are usually treated with radical resection surgeries, and high-grade tumors need definitive radiotherapy in addition to radical resection surgeries and neck dissection [1,7,8]. Conservative treatment measures are usually associated with a 40% recurrence rate as compared to radical surgeries (13%) [2,9]. Metastasis is usually local and late and is seen in about 12% of cases [2,10]. There are only a few case reports in the literature mentioning the intraosseous involvement of MEC [1,4,5]. This manuscript highlights the clinical presentation and management of two cases of intraosseous MEC of the mandible.

## Case presentation

Case 1

**Figure 1:**
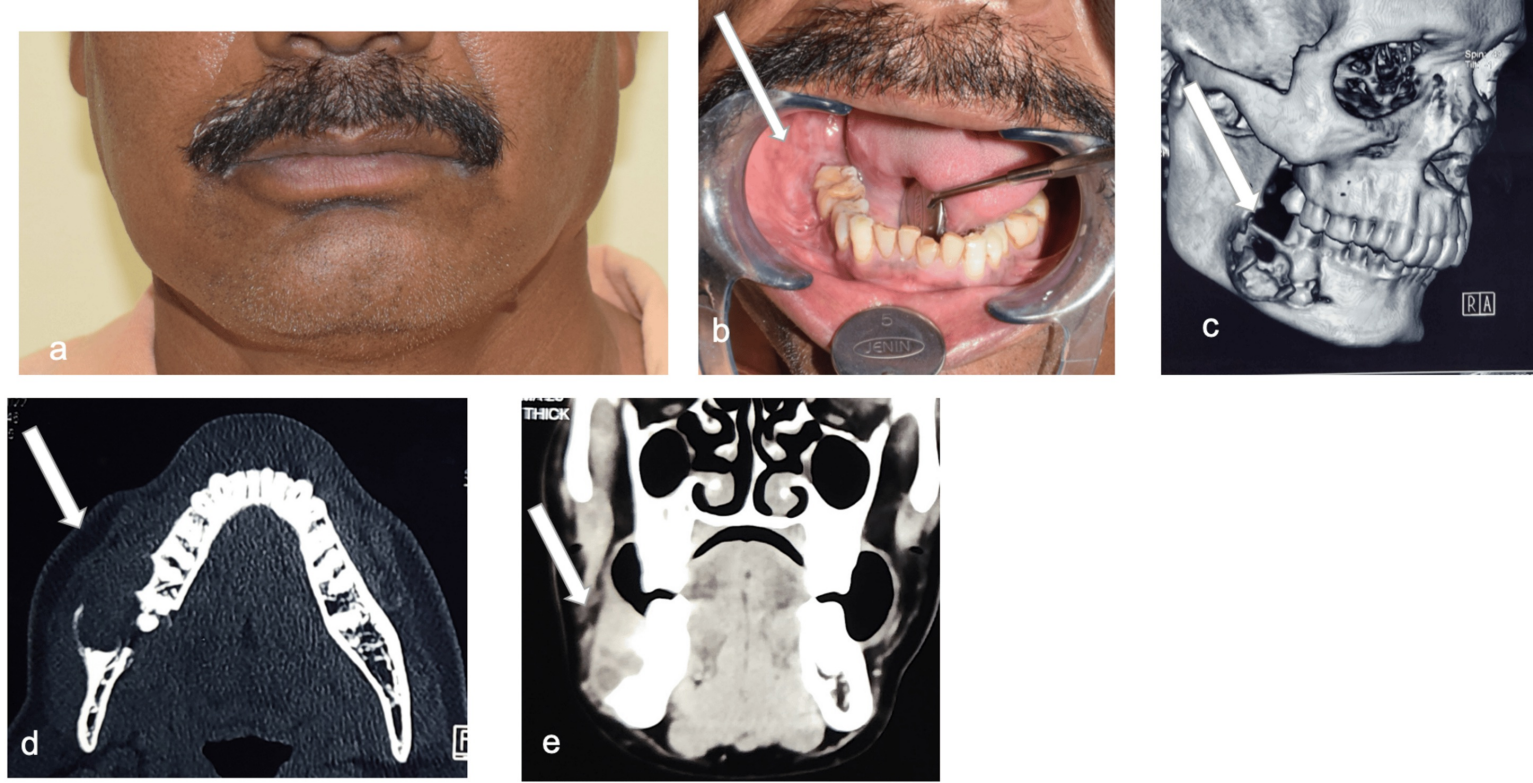
a: Extraoral image. b: Intraoral image. c: CECT showing an osteolytic lesion. d. Axial section. e. Coronal section. CECT: Contrast-enhanced computed tomography

**Figure 2:**
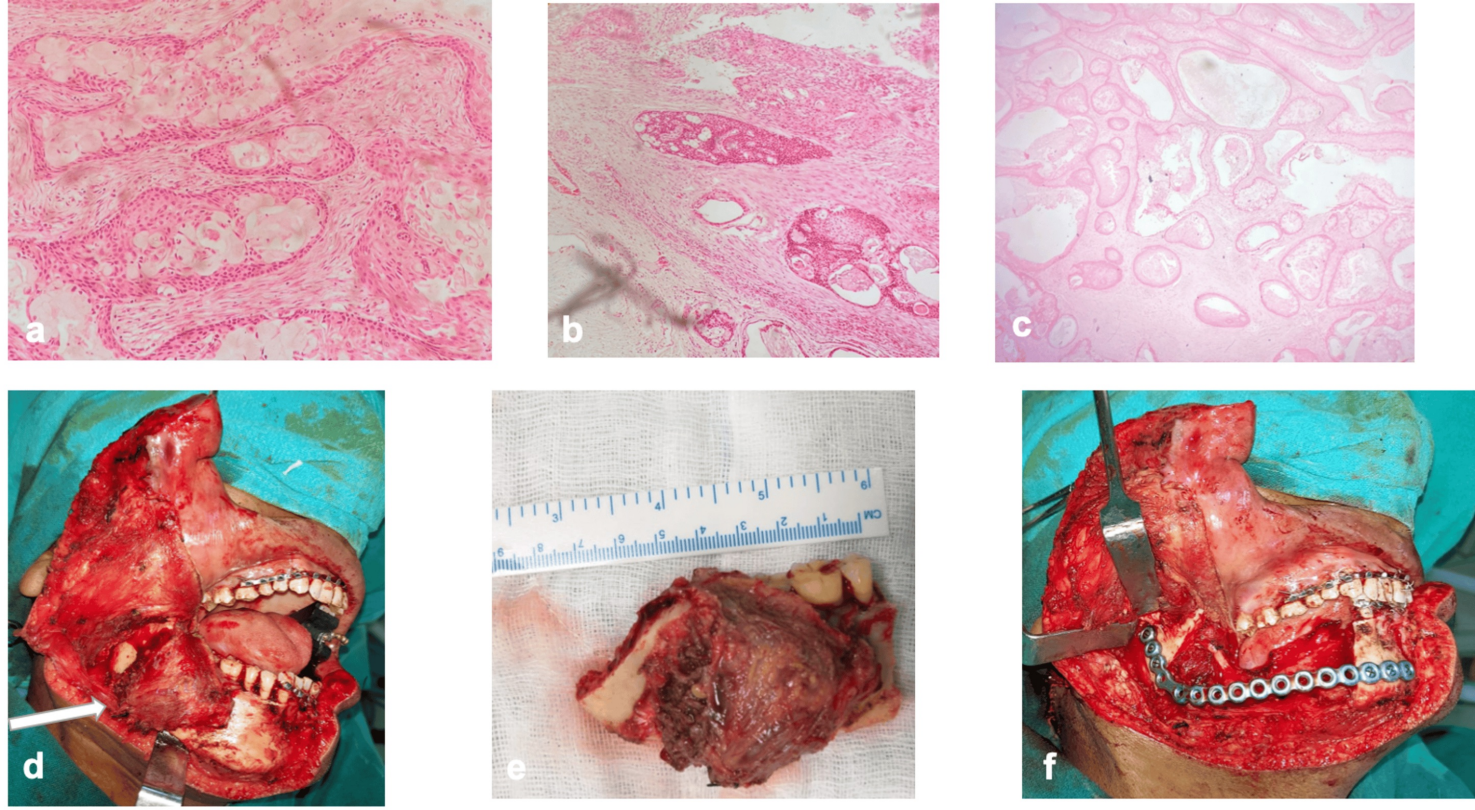
a: Epidermoid cells (H&E stain, high power). b: Nests of epidermoid cells (H&E stain, low power). c: Mucous cells lining glandular spaces. d: Intraoperative image showing tumor mass. e. Tumor specimen. f. Reconstruction done with 2.8mm recon plate

**Figure 3:**
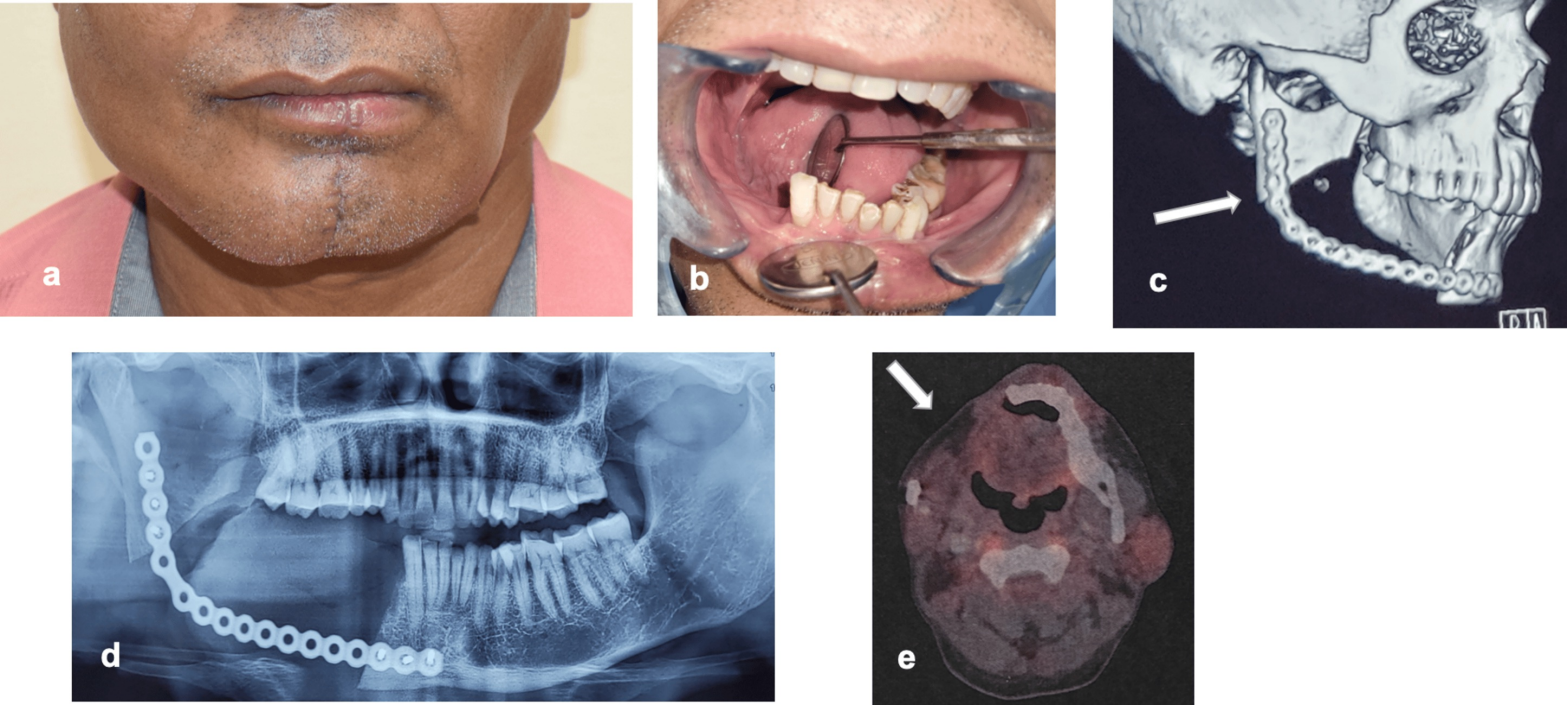
a: Postoperative extraoral image. b: Postoperative intraoral image. c: Postoperative 3D CT image. d: Postoperative OPG. e: Postoperative PET scan OPG: Orthopantomogram

A 45-year-old male patient with a medical history of hypertension reported to Department of Dentistry, AIIMS Bhubaneswar with a complaint of painless swelling on the right-side lower back teeth region. He was normal six months back and noticed a painless, slow-growing swelling in the right-side lower back teeth region. On extra-oral examination, there was a painless, firm, non-fluctuant, and non-pulsatile diffuse swelling of 2cm × 2cm in size on the right-side mandibular angle region, causing mild facial asymmetry (Figure 1a). On intra-oral examination, there was a mild erythematous lesion in the buccal vestibule extending from the mesial aspect of the right mandibular 2nd molar to the retro-molar area distally. There was a pathological migration of the 2nd and 3rd mandibular molars along with ulceration over the gingiva (Figure 1b). There was no cervical lymphadenopathy found. In contrast-enhanced computerized tomography (CECT), a well-defined osteolytic lesion of approx. 3cm × 2cm with mildly enhancing soft tissue lesion was found in the right-side body and angle regions of the mandible resembling an odontogenic cyst or tumor (Figures 1c-1e). The incisional biopsy was done, and a histopathological picture showed the presence of mucinous, intermediate, and epidermoid cells within the tumor on hematoxylin and eosin (HE) stained sections, confirming the diagnosis of intermediate-grade mucoepidermoid carcinoma (Figures 2a-2c). The patient was operated on under general anesthesia, and access to the lesion was made by a right submandibular incision with a lip split approach. A composite segmental resection of the tumor mass enclosed within the masseter muscle laterally and medial pterygoid muscle medially was done from the right-side mandibular 1st premolar to half of the ramus region with a 1.5cm bone and 1cm soft tissue safety margin. The lingual nerve was preserved; however, the right inferior alveolar nerve was sacrificed. The reconstruction was done with a 2.8mm reconstruction plate (Stryker Leibinger GmbH &C0.KG Germany) with respect to the defect (Figures 2d-2f). The tumor mass sent for histopathological evaluation revealed an intermediate-grade mucoepidermoid carcinoma. Postoperatively, the patient was sent for radiotherapy to decrease the chances of recurrence and metastasis. A radiation dose of 60 grays at the rate of 2 grays per fraction over two and a half months was given. The patient was followed every three months for a period of one year with no clinical features of recurrence, which was confirmed on the PET scan after one year of follow-up (Figures 3a-3e).

Case 2 

**Figure 4:**
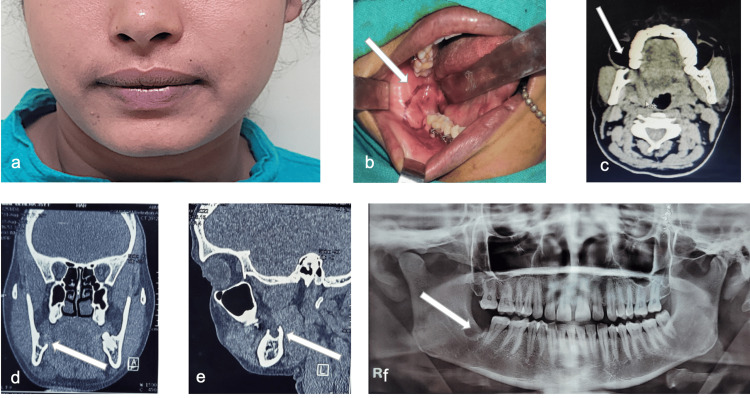
a: Extra oral image. b: Intraoral image. c-e: Axial, coronal, and sagittal CECT sections showing an osteolytic lesion. f: Preoperative OPG CECT: Contrast-enhanced computed tomography; OPG: orthopantomogram

**Figure 5:**
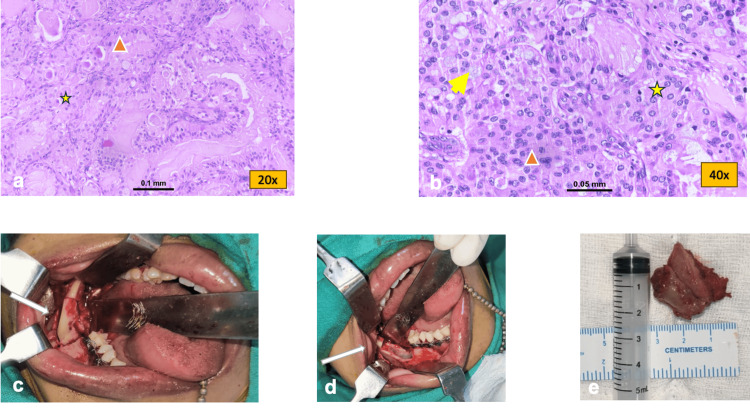
a and b: Hematoxylin and eosin (HE) stained section showing mucinous cells (yellow arrow), intermediate cells (orange triangle) and epidermoid cells (yellow star) within the tumor. c and d: Intraoperative image showing resection of lesion. e: Resected tumor specimen

A 40-year-old female patient reported to Department of Dentistry AIIMS Bhubaneswar with a complaint of mild pain in the right-side lower back teeth region. She was normal eight months back and noticed a slow-growing swelling in the right-side back teeth region. On extra-oral examination, there was no gross facial asymmetry (Figure 4a). On intra-oral examination, there was a firm swelling of about 1cm × 1cm in size present in the buccal vestibule extending from the distal aspect of the mandibular 2nd molar to the retro-molar area. The swelling was extending lingually towards the retromolar gingiva with respect to the distal aspect of mandibular 2nd molar (Figure 4b). There was no cervical lymphadenopathy. On panoramic radiograph, a unilocular radiolucent lesion was found in the right-side mandibular 3rd molar region (Figure 4f). On CECT, a well-defined unilocular osteolytic lesion of approximately 1.5cm ×1cm size with mild enhancing soft tissue lesion was found in the region of the mandible 3rd molar socket resembling an odontogenic cyst or tumor (Figures 4c-4e). Incisional biopsy was done under local anesthesia from the affected area. The mucinous cells, intermediate cells, and epidermoid cells within the tumor on hematoxylin and eosin (HE) stained sections were seen in the histopathological images that confirmed the intermediate-grade IMEC as the final diagnosis (Figures 5a, 5b).

**Figure 6:**
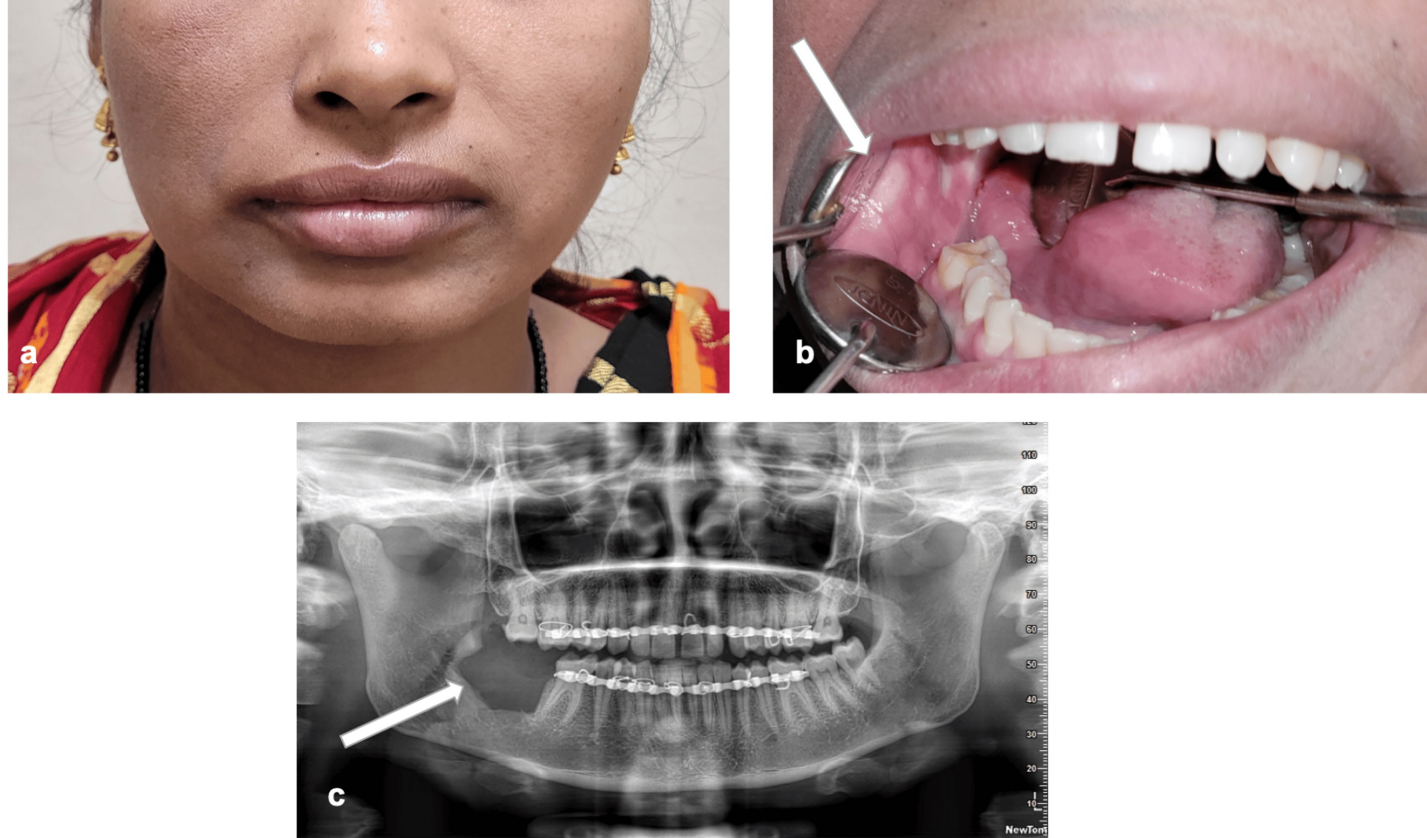
a and b: Extraoral and intraoperative postoperative images. c: Postoperative OPG OPG: Orthopantomogram

The patient was operated under general anesthesia, and access to the lesion was made by intraoral approach. The right mandibular second molar was extracted and wide local incision of the tumor mass with 1.5cm safe bony and 1cm soft tissue margin was done from the socket of 2nd molar and posteriorly extending to the retro-molar area. The right-side lingual nerve and inferior alveolar nerve were preserved (Figures 5c-5e). The postoperative histopathology report revealed the involvement of the medial margin of the tumor mass. Thus, adjuvant radiation therapy was considered, and a radiation dose of 60Gy at the rate of 2 grays per fraction over two and half months was given to prevent recurrence. The patient was followed every three months for a period of one and a half years, and there was no recurrence of the tumor till the end of the one and half-year follow-up (Figures 6a-6c). 

## Discussion

MEC is the most common malignant tumor (5% to 10%) of all salivary gland tumors [2]. The intraosseous MEC comprises only 2% to 3% of all head and neck MECs [2,11-13]. It is usually found twice in the mandible compared to the maxilla and is more frequently present in the premolar and molar regions of the mandible. It can be seen in all age groups, with predominance in the 4th and 5th decades of life without sex predilection. In children, it is very rare, thus indicating that it is not a developmental disturbance or teratoma [2,13]. In puberty, the incidence of MEC is high, indicating the important role of hormones [13]. The exact etiology of MEC has yet to be discovered. Entrapped embryonic remnants of salivary gland tissue within bones, an extension of mucosal and submucosal glands into maxillary sinuses, and transformation of odontogenic cystic lining into these lesions are common etiology of intraosseous MEC [1,2,5,6]. Recently, Bouquet et al. in the year 2000 found the presence of salivary gland tissue in 0.3% of specimens of maxillary bones [6]. The common symptoms of intraosseous MEC are pain and swelling with rare features of tooth mobility and paresthesia [1,2].

The differential diagnosis for a mixed radiolucent/radio-opaque mandibular lesion in tooth-bearing areas encompasses several categories (a) fibro-osseous lesions (cemento-osseous dysplasia), (b) odontogenic (dentigerous and radicular cysts) and non-odontogenic cysts (aneurysmal bone cyst), (c) infectious and inflammatory lesions, and (d) benign or malignant neoplasms (either odontogenic, non-odontogenic, or metastatic) [1]. Ill-defined lesions are more indicative of malignancy due to the potential for tissue infiltration and destruction. In contrast, benign processes usually result in the displacement of normal anatomy, leading to well-defined borders on imaging studies. Radiologically, these tumors represent diverse presentations, usually as unilocular or multilocular radiolucency [4]. These tumors sometimes appear as scalloped lesions with well-defined margins. The mixed radiolucent-radiopaque lesions have also been reported in the literature [4,5]. Chan et al. noted that the well-delineated sclerotic margins with amorphous sclerotic bone and many locations are the common radiological features of MEC; even root resorption along with tooth mobility is found in some cases [4,14-16]. Chan et al. also observed that all their cases appeared as unilocular or multilocular bony radiolucency and resembled different benign and malignant odontogenic jaw lesions [16]. Our first case appeared as a multilocular radiolucent lesion with locations, and the second case appeared as a well-defined unilocular radiolucent lesion resembling the odontogenic cyst. The presence of amorphous materials, along with the features of malignancy, helps in the differentiation of these lesions from other odontogenic lesions [4]. Eversole et al. noted that 50% of these tumors had a relationship with odontogenic cysts and tumors [11]. However, in our cases, such a relation was not found. Similar to our cases, Brookstone et al. also did not find such a relationship [9]. Thus, histopathological examination is the gold standard for the diagnosis of these types of lesions. Histopathological examination reveals islands and nests of intermediate, mucous, and epidermoid cells lying in various-sized cystic spaces in the fibrous connective tissues [4]. Most cases of the central MEC reported in the literature were of low-grade tumors similar to our two cases [1,2]. Pires et al. observed that glandular odontogenic cysts also share similar histological features and molecular essays may be required to differentiate MEC from other similar lesions [4,17]. Sometimes, incorrect diagnosis of the lesion may lead to overtreatment and undue complications for patients. Pires et al. observed the expression of cytokeratin 5,7,8,14,18 in central MEC and cytokeratin 5,7, 8,13,14,19 in glandular odontogenic cysts (GOC). They also found different percentages of expression of cytokeratin 18 (100 MEC versus 30% in GOC) and cytokeratin 19 (100 MEC versus 30% in GOC). Thus, cytokeratins could be used for the differentiation between these two lesions [17]. Khan et al. also found the presence of TORC1/MAML2 gene expression in central MEC cases [18]. 

The most commonly accepted criteria for diagnosis of MEC proposed by Alexander et al. and modified by Browand and Waldron include intact bony cortical plates on CT, evidence of bony destruction on radiographs, other primary tumor exclusion that mimics the central tumor histologically, exclusion of an odontogenic tumor, histopathologic confirmation and intracellular mucin detection [9,14,19]. Our first case had cortical bone expansion with the presence of buccal cortex perforation. Thus, a thorough investigation was carried out to exclude the metastasis of any other lesion. However, the histopathological features were typical of the lesion with the presence of intracellular mucin. Our second case was an unilocular lesion without cortical erosion. 

The treatment of intraosseous MEC depends upon the size of the tumor, microscopic grading, staging, clinical lymphadenopathy, post-surgical margin involvement, and perineural invasion [1,2,9]. Various conservative procedures like enucleation, curettage, marsupialization, and marginal resection with or without adjuvant therapy are associated with a 40 % relapse rate [1,2,9]. Hence radical treatment options like segmental resection, en bloc resection, and hemimandiblectomy with or without adjuvant therapy are the gold standard treatment options. Intermediate and high-grade need treatment with adjuvant radiotherapy [2]. Patients having cervical lymphadenopathy need neck dissection [20]. Our 2nd case was a low-grade tumor without cervical lymphadenopathy. Thus, segmental resection without neck dissection was considered a standard treatment. However, our 1st case was of intermediate grade tumor without neck involvement so postoperatively radiotherapy was considered as standard treatment. Long-term follow-up of these cases should be considered as there may be a possibility of recurrences and regional metastasis.

## Conclusions

Although it is a rare neoplasm, IMEC is the most common and well-recognized intraosseous salivary gland tumor. Local tumor recurrence is the main cause of death in these tumors. The present cases show that the clinical significance of these tumors should never be underestimated, emphasizing the importance of radical treatment, adjuvant therapy, and a careful histopathological evaluation of all excised tissues, so that such neoplastic transformation can be effectively identified and treated.

## References

[1] Abt NB, Lawler ME, Zacharias J, Lahey ET (2019). Primary intraosseous mucoepidermoid carcinoma of the mandible: radiographic evolution and clinicopathological features. BMJ Case Rep.

[2] Simon D, Somanathan T, Ramdas K, Pandey M (2003). Central mucoepidermoid carcinoma of mandible - a case report and review of the literature. World J Surg Oncol.

[3] Stewart FW, Foote FW, Becker WF (1945). Muco-epidermoid tumors of salivary glands. Ann Surg.

[4] Moghadam SA, Moghadam FA (2014). Intraosseous mucoepidermoid carcinoma: report of two cases. J Dent (Shiraz).

[5] Johnson B, Velez I (2008). Central mucoepidermoid carcinoma with an atypical radiographic appearance. Oral Surg Oral Med Oral Pathol Oral Radiol Endod.

[6] Bouquot JE, Gnepp DR, Dardick I, Hietanen JH (2000). Intraosseous salivary tissue: jawbone examples of choristomas, hamartomas, embryonic rests, and inflammatory entrapment: another histogenetic source for intraosseous adenocarcinoma. Oral Surg Oral Med Oral Pathol Oral Radiol Endod.

[7] Bell D, Lewis C, El-Naggar AK, Weber RS (2016). Primary intraosseous mucoepidermoid carcinoma of the jaw: reappraisal of The MD Anderson Cancer Center experience. Head Neck.

[8] Moss WJ, Coffey CS, Brumund KT, Weisman RA (2016). What is the role of elective neck dissection in low-, intermediate-, and high-grade mucoepidermoid carcinoma?. Laryngoscope.

[9] Brookstone MS, Huvos AG (1992). Central salivary gland tumors of the maxilla and mandible: a clinicopathologic study of 11 cases with an analysis of the literature. J Oral Maxillofac Surg.

[10] Lebsack JP, Marrogi AJ, Martin SA (1990). Central mucoepidermoid carcinoma of the jaw with distant metastasis. A case report and review of the literature. J Oral Maxillofac Surg.

[11] Eversole LR (1970). Mucoepidermoid carcinoma: review of 815 reported cases. Oral Surg Oral Med Oral Pathol.

[12] Gingell JC, Beckerman T, Levy BA, Snider LA (1984). Central mucoepidermoid carcinoma: review of literature and report of a case associated with an apical periodontal cyst. Oral Med Oral Surg Oral Pathol.

[13] Ezsias A, Sugar AW, Milling MA, Ashley KF (1994). Central mucoepidermoid carcinoma in a child. J Oral Maxillofac Surg.

[14] Waldron CA, Koh ML (1990). Central mucoepidermoid carcinoma of the jaws: report of four cases with analysis of the literature and discussion of the relationship to mucoepidermoid, sialodontogenic, and glandular odontogenic cysts. J Oral Maxillofac Surg.

[15] Zhou CX, Chen XM, Li TJ (2012). Central mucoepidermoid carcinoma: a clinicopathologic and immunohistochemical study of 39 Chinese patients. Am J Surg Pathol.

[16] Chan KC, Pharoah M, Lee L, Weinreb I, Perez-Ordonez B (2013). Intraosseous mucoepidermoid carcinoma: a review of the diagnostic imaging features of four jaw cases. Dentomaxillofac Radiol.

[17] Pires FR, Chen SY, da Cruz Perez DE, de Almeida OP, Kowalski LP (2004). Cytokeratin expression in central mucoepidermoid carcinoma and glandular odontogenic cyst. Oral Oncol.

[18] Khan HA, Loya A, Azhar R, Din NU, Bell D (2010). Central mucoepidermoid carcinoma, a case report with molecular analysis of the TORC1/MAML2 gene fusion. Head Neck Pathol.

[19] Alexander RW, Dupuis RH, Holton H (1974). Central mucoepidermoid tumor (carcinoma) of the mandible. J Oral Surg.

[20] He Y, Wang J, Fu HH, Zhang ZY, Zhuang QW (2012). Intraosseous mucoepidermoid carcinoma of jaws: report of 24 cases. Oral Surg Oral Med Oral Pathol Oral Radiol.

